# The low invasiveness design (LID) flap in immediate implant placement: a 20-patient case series on a novel flap design based on new insights into papilla vascularization

**DOI:** 10.1007/s00784-025-06496-x

**Published:** 2025-08-12

**Authors:** D. De Santis, S. Wang, P. Montagna, P. Faccioni, A. Zangani, F. Balliu, F. Melloni, F. Gelpi

**Affiliations:** 1 Head and Neck Department, Department of Surgery, Dentistry, Pediatrics and Gynecology, University of Verona, Verona, Italy; 2 Department of Dental Implantology, The Affiliated Stomatological Hospital, Zunyi Medical University, Zunyi, Guizhou China

**Keywords:** Immediate implant placement, Esthetic zone, Dental implants, Surgical technique

## Abstract

**Objectives:**

This study presents a novel Low Invasiveness Design (LID) flap for Immediate Implant Placement (IIP) and evaluates its clinical and esthetic outcomes in a 20-patient retrospective case series. The LID flap is based on recent insights into papilla vascularization and aims to balance the benefits of flapless and conventional flap techniques.

**Materials and methods:**

Twenty patients requiring IIP in the anterior maxilla were treated with the LID flap. The study included non-smoking, systemically healthy individuals. Esthetic outcomes and patient satisfaction were assessed one year after final prosthetic rehabilitation respectively using the Pink Esthetic Score (PES) and the Visual Analogue Scale (VAS).

**Results:**

No implant failures or major complications were reported. A minor case of flap distress (5%) was observed and healed spontaneously without intervention. The mean PES was 12.35 ± 0.99, indicating satisfactory esthetic outcomes, while the mean VAS score was 9.15 ± 0.75. The flap design successfully preserved papilla height and granted soft tissue stability.

**Conclusions:**

The LID flap presents a promising approach for IIP, preserving soft tissue esthetics while ensuring surgical precision. The preliminary results suggest that this technique may provide an alternative to traditional flap designs. *Clinical Relevance*: The LID flap may serve as an alternative to traditional incision designs in IIP procedures.

**Supplementary Information:**

The online version contains supplementary material available at 10.1007/s00784-025-06496-x.

## Introduction

Dental implants have become a safe and reliable solution for replacing missing teeth. In the early years, clinicians primarily focused on achieving osseointegration, using bone as the key reference for implant positioning. Today, osseointegration is a predictable and reproducible process, shifting the focus toward long-term success, primarily based on precise prosthetic positioning, optimal esthetics, and stable soft tissue integration. Alongside these advancements, patients’ perceptions of implant treatment have also evolved. The rise of social media has played a crucial role in shaping expectations and increasing awareness [1]. The concept of the “social smile”, which highlights the importance of the upper anterior teeth’s visibility during smiling, has heightened both esthetic and functional demands. Patients now seek flawless results in the shortest time possible. Despite IIP’s growing popularity, awareness of its potential complications remains limited.

To address these growing demands, faster placement and loading protocols for dental implants have been developed, with a particular emphasis on implants in the esthetic zone. According to the Consensus Report of the International Team for Implantology (ITI) [2], Immediate Implant Placement (IIP) is performed on the same day as tooth extraction, while Early Implant Placement (EIP) occurs during soft tissue or partial bone healing, distinguishing it from Conventional or Late Implant Placement (LIP). When an implant is placed immediately after extraction and prosthetically loaded on the same day, this is classified as a Type 1 A loading protocol. Given the increasing esthetic expectations, patients are often reluctant to delay implant placement following tooth extraction. Many prefer immediate provisionalization along with implant placement to maintain esthetics and function throughout the healing period.

Immediate Implant Placement (IIP) requires specific criteria to be met to achieve high success rates. According to the SAC Classification (Straightforward (S), Advanced (A), and Complex (C)), this procedure is always categorized as at least advanced [3]. Key parameters for success include a fully intact buccal bone wall of at least 1 mm thickness and a thick gingival biotype, along with adequate 3D bone volume apically or palatally to ensure sufficient primary stability, especially when a Type 1 A loading protocol is used [4]. However, achieving these optimal conditions in the anterior maxilla is particularly challenging. Braut et al. [5] reported that only 4.6% of upper central incisor sites had a thick facial bone wall at the cementoenamel junction, while most patients in the same region exhibited a thin gingival biotype [6]. Further complicating IIP are the precise requirements for implant positioning, including bucco-palatal placement, implant depth, angulation, and interproximal relationships [7, 8]. Given that IIP is inherently aimed at minimizing invasiveness while maximizing esthetic outcomes, it is frequently performed using a flapless technique [9]. This approach is recommended as it preserves the supraperiosteal blood supply, reducing the risk of mid-facial recession while also minimizing soft tissue trauma and scarring.

In the daily clinical practice those criteria are rarely satisfied, but nevertheless patients desire IIP. In such cases a flap procedure is advised both to facilitate correct implant placement and to perform concomitant hard and soft tissue enhancing procedures. The 6th EAO Consensus Conference [10] highlighted that soft tissue augmentation during Immediate Implant Placement (IIP) yields enhanced esthetic outcomes. Notably, such augmentation in the esthetic zone has been associated with a reduced recession of the mid-buccal mucosa compared to cases without soft tissue augmentation. A systematic review by Seyssens et al. [11] on hard tissue management suggests that socket grafting in the context of IIP enhances both soft and hard tissue stability, effectively minimizing midfacial recessions in the premaxilla. This is especially pertinent in cases where the buccal bone wall is less than 1 mm thick.

When a flap procedure is devised, several incision design can be considered [12]. Literature reports that scenarios requiring extensive hard tissue grafting are better handled by conventional full-thickness flaps, while papillae-sparing techniques can be used when limited augmentation is required. These flap designs are better at preventing papilla atrophy if compared to intrasulcular incisions at second stage surgery [13], and their use is advocated to preserve the height of the papillae during implant placement in the esthetic zone [14]. Papillae-sparing flaps can mitigate the invasiveness of IIP while allowing for a better sight of the surgical field when compared to flapless procedures, but they still require vertical buccal incisions that might compromise blood supply according to recent papillae vascularization concepts [14, 15]. An alternative approach is the envelope flap, which eliminates the need for vertical releasing incisions but involves a papilla-splitting incision, which may still compromise interproximal vascularization and pose a risk to papilla preservation.

In this context, the present preliminary retrospective case series evaluates the esthetic outcomes of a novel flap design for Immediate Implant Placement (IIP) on 20 patients. This design, called LID (Low Invasiveness Design), is based on recent studies investigating the blood supply of the gingiva in the premaxilla [15–17], and was developed to address key limitations of existing techniques: while papillae-sparing incisions help prevent soft tissue atrophy, they still require vertical buccal incisions, that may compromise papillae vascularization. Conversely, flapless approaches, though minimally invasive, often lead to limited surgical access and uncontrolled soft tissue remodelling.

## Materials and methods

The present case series was conducted according to the 2023 PROCESS Guidelines for Case Reports in Surgery [18]. This study was conducted in accordance with the Declaration of Helsinki (2024 revision) and was reviewed by the Ethics Committee (CET Area Sud Ovest Veneto), which acknowledged the study but did not require formal approval due to its retrospective nature. Nevertheless, all patients provided written informed consent prior to participation. All patient information collected were anonymized.

### Study design

This study is presented as a retrospective case series conducted on 20 patients seeking Immediate Implant Placement (IIP) in the esthetic zone, specifically from tooth #1.4 to tooth #2.4. All patients were treated using the Low-Invasiveness Design (LID) flap at the Department of Dentistry and Maxillofacial Surgery, AOUIVR, Verona, Borgo Roma. The inclusion period spanned from January 2022 to December 2023. During this time, 52 patients underwent IIP procedures utilizing the LID flap in the esthetic zone at our institution. Of these, only 20 patients met al.l predefined inclusion criteria, completed the required clinical and prosthetic follow-up, and provided written informed consent for publication. Specifically, 15 patients were heavy smokers (≥ 10 cigarettes per day), 2 had poorly controlled diabetes, 4 presented with anatomical defects incompatible with the inclusion criteria, and for 11 patients, either CBCT data were unavailable, follow-up was incomplete, or publication consent was not granted. A retrospective case series was selected as an exploratory study to assess the clinical feasibility and esthetic outcomes of the LID flap, providing a foundation for future controlled trials. Esthetic outcomes and patient satisfaction were evaluated one year after final restoration delivery using the Pink Esthetic Score (PES) [19–21] and the Visual Analogue Scale (VAS) [22, 23]. PES was selected as the primary outcome measure due to its clinical relevance for assessing peri-implant soft tissue esthetics, particularly in the anterior maxilla. Patient satisfaction was assessed using a VAS ranging from 0 (completely unsatisfied) to 10 (extremely satisfied). Relevant clinical data, including socket integrity and visually assessed gingival phenotype [24], were obtained from patient records as assessed by an experienced surgeon (Prof. D. De Santis).

### Data and statistics

All statistical analyses were conducted using Stata 16 (StataCorp, College Station, TX, USA). Descriptive statistics were calculated for all relevant variables, including means, standard deviations, and frequency distributions. The significance level was set at *p* < 0.05 for all inferential tests.

### Inclusion and exclusion criteria

Inclusion criteria were patients undergoing IIP in the esthetic zone (tooth #1.4 to #2.4), with standard oral hygiene and no active periodontitis in the neighbouring teeth. Furthermore, the neighbouring teeth needed to be correctly aligned into occlusion and do not present major restorative concerns. Additionally, availability of preoperative CBCT data was mandatory for inclusion. Exclusion criteria included active apical periodontitis on the extracted tooth, a contraindication to IIP [25], heavy smoking (≥ 10 cigarettes per day), uncontrolled diabetes (HbA1c ≥ 7.5%), a history of alcoholism or chronic drug abuse, predisposing factors for antiresorptive agent-related osteonecrosis of the jaw (ARONJ), local radiation therapy within the preceding five years, and any form of immunosuppression. Patients with a thin gingival biotype or a small, favourable V-shaped bone dehiscence were not excluded. However, cases presenting unfavourable bone defects requiring more than socket preservation with a membrane without fixation or extending beyond the coronal third of the alveolar process were not eligible for inclusion. Specifically, only defects classified as ST3 Subclass A (ST3A) according to Steigmann et al. were eligible for inclusion [26].

### Flap design overview

#### Clinical presentation

**Fig. 1:**
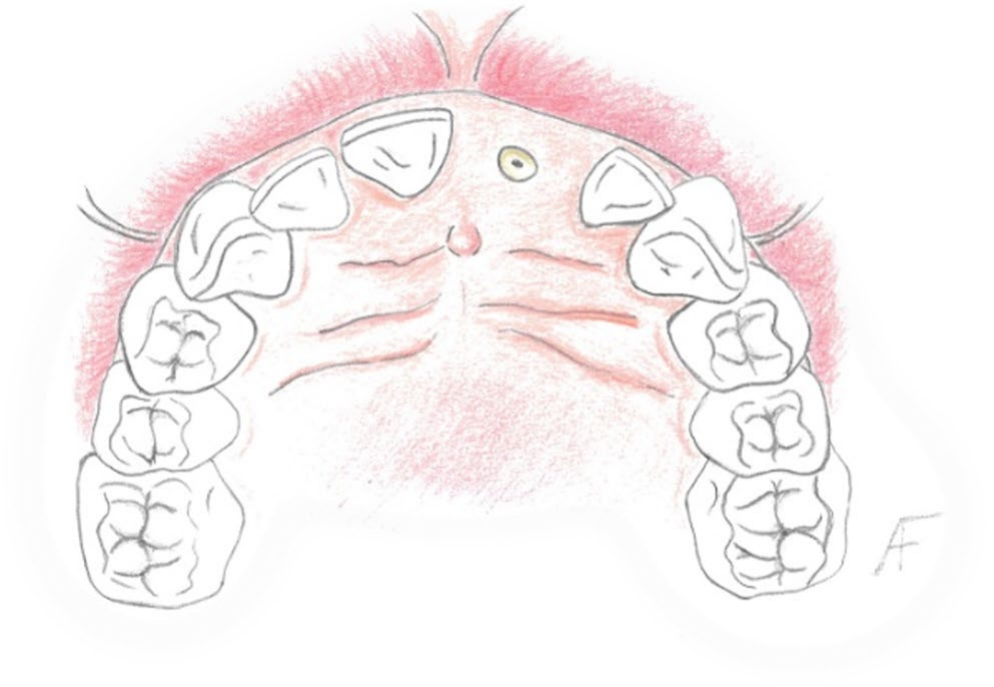
Schematic representation of the initial condition of a typical candidate for the LID technique. In this case, tooth #2.1 has deteriorated to a root remnant; however, the same principles apply if a clinical crown is still present

The usual scenario in which the authors advise this technique starts with a failing tooth in the esthetic zone, often lacking much of its clinical crown (Fig. 1). In cases presenting a mostly intact clinical crown the initial step involves performing a clinical crown reduction. This is achieved using a thin, flame-shaped diamond bur (KOMET 862.314.010, KOMET Italia, Verona, Italy). The reduction of the clinical crown is primarily focused on its incisal edges and care is also taken to ensure that any contact point is eliminated.

#### Sindesmotomy prior to flap elevation

**Fig. 2:**
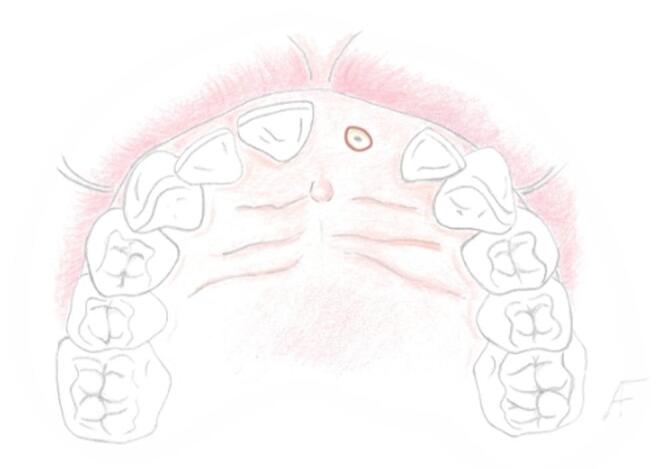
Sindesmotomy

A complete sectioning of the periodontal ligament surrounding the compromised tooth (Fig. 2) is performed. A microsurgical beaver scalpel is preferably used, inserted at an oblique angle into the gingival tissue to carefully navigate around any overgrown soft tissue covering the tooth’s socket. Once the blade’s tip is properly positioned at the socket’s boundary, realign the blade to become parallel with the axis of the socket. Proceed with the incision, applying controlled depth based on the resistance offered by the socket walls, performing a bone sounding with the scalpel itself.

#### Initial vestibular incision

The initial vestibular incision is made using the same microsurgical beaver blade. It is a horizontal full-thickness incision along the gingival margin of one of the two adjacent teeth, typically beginning at the vestibular midpoint of the proximal (forward-facing) tooth next to the failing one. The incision remains full-thickness up to the tooth’s medial transition line (line angle) before transitioning to a split-thickness incision in the interproximal area. Depending on procedural needs, the surgeon may extend the incision further to include additional teeth.

#### Interproximal incision

Continuing from the initial incision, as the surgical approach reaches the interproximal area, the incision becomes split thickness. This cut extends across the alveolar ridge, from the vestibular side toward the palatal, carefully tracing the contour of the interproximal toot. This technique is deliberately chosen to conserve as much of the papillary vascularization as possible. In cases of thin gingival biotypes, the incision can be performed as a full-thickness flap, minimizing the risk of flap perforation. This method still provides adequate blood supply to the papillae by avoiding mesio-distal (horizontal) incisions.

#### Initial palatal incision

Continuing from the previous step, the incision advances to the palatal aspect of the adjacent tooth, reverting to a full-thickness approach. Here, the surgical strategy mirrors the incision delineated in phase. The surgeon extends the incision along the palatal surface, ensuring an intrasulcular incision that follows the gingival margin, culminating at the palatal midpoint of the tooth.

#### Vertical palatal incision

Upon reaching the palatal midpoint, a vertical releasing incision is made. This incision should extend towards, but not include, the natural palatine rugae. The incision should aim to halt before directly involving the rugae and usually extends for 4–5 mm maximum. The authors suggest, as an additional calibration reference, that this incision can be vertically extended up to half the vertical extent of the incisive papilla.

#### Horizontal palatal incision

After achieving the appropriate height with the vertical palatal incision, a horizontal incision is carried out. This incision runs horizontally from the apical point of the vertical incision to a point directly across, mirroring the midpoint of the palatal aspect of the contralateral tooth. The horizontal incision’s ending should be aligned at the same vertical level as the starting point. Halfway through this horizontal pathway there is the risk of encountering the nasopalatine canal. To circumvent this scenario and the associated sequelae, the incision adopts a semicircular trajectory, arcing around the incisive papilla.

#### Completion of flap design

**Fig. 3:**
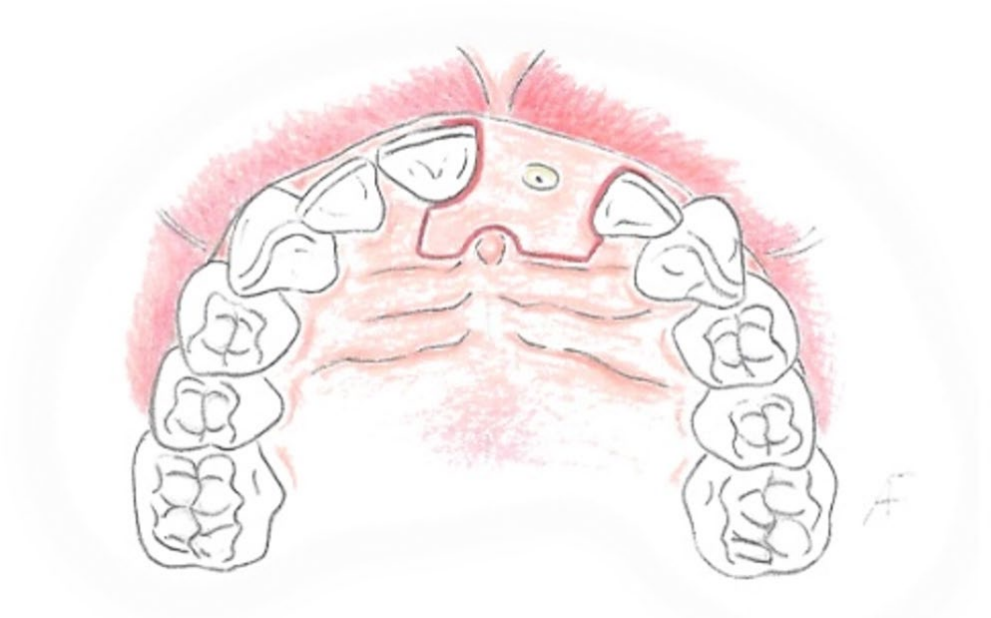
The flap design is completed

In finalizing the flap design, the procedure entails replicating the incisions symmetrically the contralateral proximal tooth (Fig. 3).

#### Flap elevation using microsurgical instruments

**Fig. 4:**
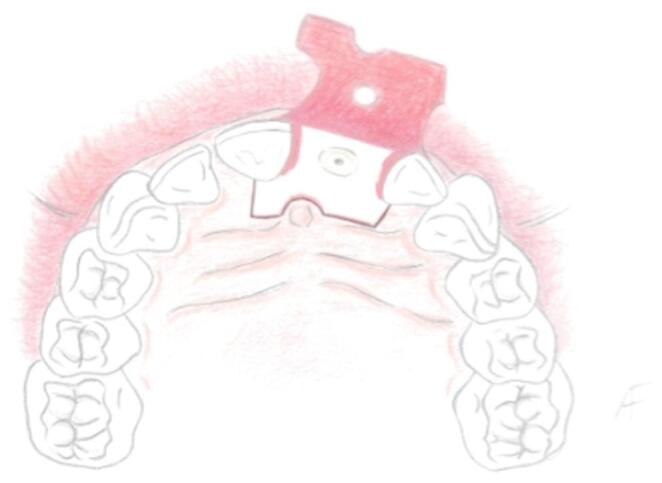
The flap is reflected on the buccal side utilising microsurgical instruments

To correctly and atraumatically handle the flap the authors suggest the use of microsurgical instruments. Utilizing a microsurgical periosteal elevator and atraumatic surgical tweezers the flap is elevated from the palatal side to the buccal aspect, slipping over the failing tooth thanks to the previously executed sindesmotomy, avoiding crestal horizontal incisions and maintaining soft-tissue integrity (Fig. 4). With the flap fully prepared, the surgical field is now arranged for atraumatic tooth extraction.

#### Atraumatic tooth extraction and implant placement

Taking advantage of visibility over the failed tooth and its socket boundaries an atraumatic extraction, in some selected cases involving the use of piezoelectric inserts to better expose the root surface, is performed. Such an approach ensures consistent preservation of alveolar bone integrity. Prosthetically driven implant placement follows.

#### Flap closure and suture technique

**Fig. 5:**
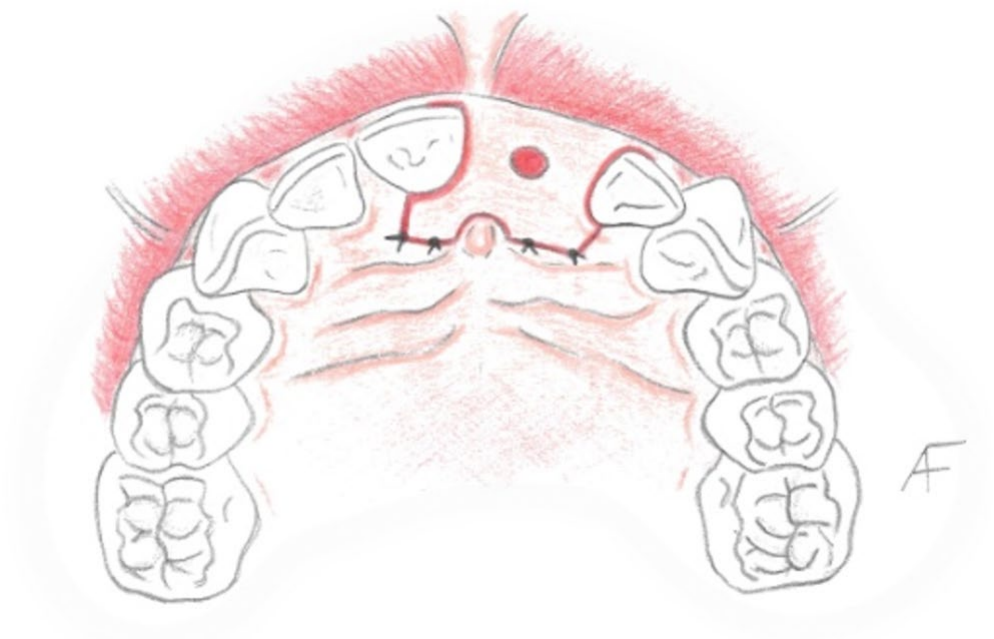
The flap is secured in place using four strategically positioned single stitches

For effective closure of the flap, simple interrupted stitches are applied using 6 − 0 Vicryl and a microsurgical needle holder. The flap design features an interlocking configuration on the palatal side. This is achieved through the palatal incision design and the semicircular incision around the incisive papilla. The resilience of the keratinized palatal mucosa and the flap design reduce the need for positional adjustments. Once the flap is gently placed in its original position it spontaneously tends to retain its placement. Key closing sutures are strategically placed at the horizontal segment of the palatal flap to secure primary intention healing *(*Fig. 5). Additional sutures may be utilized as required for haemostasis and to ensure a hermetic closure.

#### Case-specific variations

The proposed flap design can be modified to accommodate a variety of clinical situations, as limited Guided Bone Regeneration (GBR) and Guided Tissue Regeneration (GTR). With regards to the latter the authors’ advised technique involves the preparation of a partial thickness envelope flap on the buccal aspect [27] and xenogeneic collagen matrix [28, 29]. Notably it is only in this context that the buccal soft tissues are subjected to visible marginal horizontal incision. Moreover, in cases of ankylosis, the buccal bone plate may be unsalvageable. In such situations, elevating the vestibular flap facilitates more extensive bone grafting, effectively converting the approach into a conventional flap with a palatal extension. While this preserves the interlock design’s advantages, it compromises some of the minimally invasive benefits. Another feasible solution in these cases would be the use of piezoelectric inserts [30].

### Surgical protocol

Patients were enrolled at the Department of Dentistry and Maxillofacial Surgery, Hospital G.B. Rossi, Verona, Borgo Roma. The protocol began with a comprehensive clinical and radiographic evaluation, including cone-beam computed tomography (CBCT). Before surgery, patients were fully informed of the potential risks and written informed consent was obtained. A digital wax-up, derived from a digital impression, was then created to guide implant positioning based on the prosthetic plan. As part of preoperative care, patients were given prophylactic antibiotic therapy consisting of Amoxicillin (875 mg) and Clavulanate (125 mg), administered as two capsules (1 g total) one hour before surgery.

On the day of surgery, strict aseptic protocols were followed. The patient rinsed with a 0.2% chlorhexidine mouthwash for 60 s, followed by skin disinfection around the oral area with 10% povidone-iodine solution. To prevent soft tissue irritation, petroleum jelly was applied to the lips and perioral tissues. Local anaesthesia was administered using 2% mepivacaine with 1:100.000 adrenaline, injected in both the upper fornix and palatal region for optimal pain control. The LID flap was designed and elevated according to the predefined protocol. Atraumatic extraction of the tooth was performed to preserve the surrounding bone and soft tissues. Socket integrity was evaluated both visually and by clinical probing. The implant platform was selected based on the tooth being replaced, with reduced-diameter implants indicated for lateral incisors and regular-diameter implants for all other teeth. Implants placed were either Implassic FT3 (DentalTech, Misinto, Italy) or NobelActive TiUltra (Nobel Biocare AB, Göteborg, Sweden), with the aim of achieving primary stability at 50 N/cm torque.

Once the implant was positioned, bone grafting was performed when the implant-to-bone gap exceeded 1.5 mm, using either Bio-Oss Collagen (Geistlich Biomaterials, Wolhusen, Switzerland), Creos xenogain (Nobel Biocare AB, Göteborg, Sweden), or SmartGraft (REGEDENT AG, Zurich, Switzerland). Additional hard and soft tissue enhancement procedures were performed based on individual patient needs, following the study protocol. At this stage, the flap was repositioned passively, ensuring tension-free adaptation before suturing. Immediately after surgery, a digital impression was acquired using scanbodies to facilitate prosthetic restoration. Within 48 h, an immediate provisional restoration was delivered. The provisional restoration was screw-retained and torqued ≥ 30 N/cm using a dynamometric wrench to ensure proper stability. Postoperative care included a six-day course of Amoxicillin (875 mg) and Clavulanate (125 mg), taken every eight hours, along with detailed oral hygiene and dietary instructions. Follow-up assessments were conducted at 3, 7, 14, and 30 days postoperatively to monitor healing and detect possible complications, as authors usually do in their everyday practice when performing IIP. At two weeks, sutures were removed, and at four months, final prosthetic rehabilitation began. One year after the procedure, esthetic outcomes and patient satisfaction were re-evaluated respectively using the Pink Esthetic Score (PES) [19] and the Visual Analogue Scale (VAS) [23].

## Results

In this retrospective case series, a total of 20 implants were placed in 12 female and 8 male participants, aged between 32 and 71 years (mean age: 56.15 ± 10.44 years). No implant failures or dropouts were recorded throughout the study. Relevant patient, site-specific and procedural characteristics are summarized (Table 1). Specifically, socket integrity (SI) was evaluated based on buccal wall integrity, as assessed on preoperative CBCT. No cases of significantly compromised buccal bone, as defined by the study’s inclusion criteria, were observed. The status of the buccal plate post-extraction was also documented. Additionally, soft tissue augmentation using an envelope flap was recorded. Hard tissue augmentation procedures were classified as either socket preservation (in sites with intact buccal walls) or guided bone regeneration (in cases involving pre-existing bony defects).

**Table 1:** Included patient characteristics. Socket integrity Ab initio (SI): intact (I), slightly compromised (SC) or considerably compromised (CC). Presence of the buccal bone plate after the extraction (BBP): present (P), partially present (PP) or Absent (A). Soft tissue augmentation procedure (STA): guided tissue regeneration (GTR) or none (NAP). Hard tissue augmentation procedure (HTA): socket preservation (SP), guided bone regeneration (GBR) or no augmentation procedure (NAP)

*N*		Sex	Age	Tooth	SI	BBP	STA	HTA
1	M		57	12	I	P	NAP	NAP
2	M		43	12	SC	P	NAP	GBR
3	M		59	12	I	P	NAP	NAP
4	F		59	12	I	P	GTR	SP
5	F		71	22	I	P	NAP	NAP
6	M		41	12	I	P	GTR	SP
7	F		32	14	I	PP	GTR	GBR
8	F		66	12	I	P	NAP	SP
9	F		56	24	I	P	NAP	SP
10	M		55	22	I	P	NAP	SP
11	F		59	22	I	P	GTR	NAP
12	M		55	11	SC	P	NAP	GBR
13	F		67	22	SC	P	GTR	GBR
14	F		57	21	I	P	NAP	NAP
15	F		38	22	SC	P	GTR	GBR
16	M		69	21	I	P	NAP	NAP
17	F		58	11	I	P	NAP	SP
18	F		66	12	SC	P	NAP	GBR
19	M		61	11	I	P	NAP	NAP
20	F		54	12	I	P	NAP	NAP

Concerning complication rates, only one patient (5%) exhibited minor superficial soft tissue distress on the 14th postoperative day. This event required no medical intervention, as spontaneous healing was observed by the end of the monitoring period. The complication was due to accidental trauma to the flap, caused by the patient chewing a hard bread crust. At the conclusion of the study, all participants achieved complete soft tissue healing, with a cumulative complication rate of 5%, exclusively linked to this isolated case of non-compliance with postoperative dietary recommendations (Table 2).

**Table 2:** Complications recorded at different times during the healing process. Only one patient exhibited flap distress during the healing period

Day	Flap dehiscence	Flap distress	Infection	Necrosis
3	0	0	0	0
7	0	0	0	0
14	0	1	0	0
30	0	0	0	0

One year after the final restoration delivery, the Pink Esthetic Score (PES) was assessed for each implant. Most implants were placed adjacent to periodontally sound natural teeth, with only four cases involving implants positioned next to another implant on one side. In the PES evaluation, the mesial and distal papillae received average scores of 1.75 (± 0.44) and 1.70 (± 0.47), respectively. The soft-tissue margin level and soft-tissue contour surrounding the prosthetic rehabilitation were rated at 1.80 (± 0.41) and 1.75 (± 0.44), respectively. The alveolar process deficiency scored 1.65 (± 0.49), while the soft-tissue colour and texture were evaluated at 1.80 (± 0.41) and 1.90 (± 0.31) respectively. These individual component scores resulted in an overall PES of 12.35 ± 0.99, to be considered an esthetic success [19]. Notably, only one patient received a score of 10 (Tables 3 and 4). A one-way ANOVA was conducted to compare PES scores across different implant sites, revealing no statistically significant differences among tooth positions (*p* = 0.730). Similar results are found for differences across different gingival biotypes (*p* = 0.900). In contrast, GBR and the presence of a bony defect prior to surgery negatively affected the PES scores (*p* < 0.001). These analyses, while informative, are exploratory in nature due to the retrospective design and limited sample size.

**Table 3:** PES score summary distinguished on a per patient basis. Diameter and length refer to the implant specifications expressed in mm. MP = Mesial papilla; dp = distal papilla; lstm = level of soft tissue margin; stc = soft tissue contour; ap = alveolar process; stcl = soft tissue color; stt = soft tissue texture; pes = pink esthetic score

*N*		Sex	Age	Tooth	Diameter	Length	MP	DP	LSTM	STC	AP	STCl	STT	PES score
1	M		57	12	3.75	10.0	2	2	2	2	2	2	2	14
2	M		43	12	3.75	11.5	2	1	2	1	2	2	2	12
3	M		59	12	3.75	10.0	1	1	2	2	2	2	2	12
4	F		59	12	3.75	10.0	2	2	2	2	2	2	2	14
5	F		71	22	3.75	13.0	1	2	2	2	2	2	2	13
6	M		41	12	3.75	10.0	2	2	2	2	1	2	2	13
7	F		32	14	4.3	10.0	2	2	2	2	2	1	1	12
8	F		66	12	3.75	10.0	2	2	2	1	2	1	2	12
9	F		56	24	4.3	10.0	2	2	1	1	2	2	2	12
10	M		55	22	3.75	11.5	2	1	1	2	2	2	2	12
11	F		59	22	3.75	10.0	2	2	2	2	2	2	2	14
12	M		55	11	4.3	11.5	2	2	2	2	1	1	2	12
13	F		67	22	3.75	10.0	2	1	2	2	1	1	1	10
14	F		57	21	4.25	13.0	2	2	1	2	1	2	2	12
15	F		38	22	3.75	10.0	1	2	2	2	1	2	2	12
16	M		69	21	4.3	10.0	2	1	2	1	1	2	2	11
17	F		58	11	4.25	13.0	1	2	2	2	2	2	2	13
18	F		66	12	3.75	10.0	2	1	1	2	2	2	2	12
19	M		61	11	4.25	10.0	2	2	2	1	1	2	2	12
20	F		54	12	3.75	11.5	1	2	2	2	2	2	2	13

**Table 4:** PES score summary distinguished on a per parameter basis

Parameter	Score 2	Score 1	Mean (± SD)
Mesial papilla	15	5	1.75 (± 0.44)
Distal papilla	14	6	1.70 (± 0.47)
Level of soft tissue margin	16	4	1.80 (± 0.41)
Soft tissue contour	15	5	1.75 (± 0.44)
Alveolar process	13	7	1.65 (± 0.49)
Soft tissue color	16	4	1.80 (± 0.41)
Soft tissue texture	18	2	1.90 (± 0.31)

Patient satisfaction was evaluated using the Visual Analogue Scale (VAS) one year after the final restoration delivery (Table 5). The VAS scores ranged from 7 to 10, with a mean score of 9.15 ± 0.75, indicating a consistently high level of satisfaction across the cohort. Among the 20 patients, 90% (18/20) reported VAS scores of 9 or 10, reflecting excellent perceived outcomes. Only two patients (10%) reported scores of 7 or 8, both of whom presented with thin gingival biotypes and cited minor esthetic concerns. The ANOVA analysis revealed a borderline significant association between VAS scores and gingival biotype (*p* = 0.054), suggesting that soft tissue characteristics may influence patient-reported satisfaction.

**Table 5:** VAS score summary distinguished based on gingival biotype

*N*	Tooth	Gingival Biotype	VAS Score
1	12	Thick	9
2	12	Thick	10
3	12	Thick	9
4	12	Thin	9
5	22	Thick	9
6	12	Thick	9
7	14	Thin	7
8	12	Thick	9
9	24	Thick	9
10	22	Thick	10
11	22	Thick	9
12	11	Thick	9
13	22	Thick	9
14	21	Thick	10
15	22	Thin	9
16	21	Thick	9
17	11	Thick	10
18	12	Thin	10
19	11	Thin	8
20	12	Thick	10

## Clinical case presentation

**Fig. 6:**
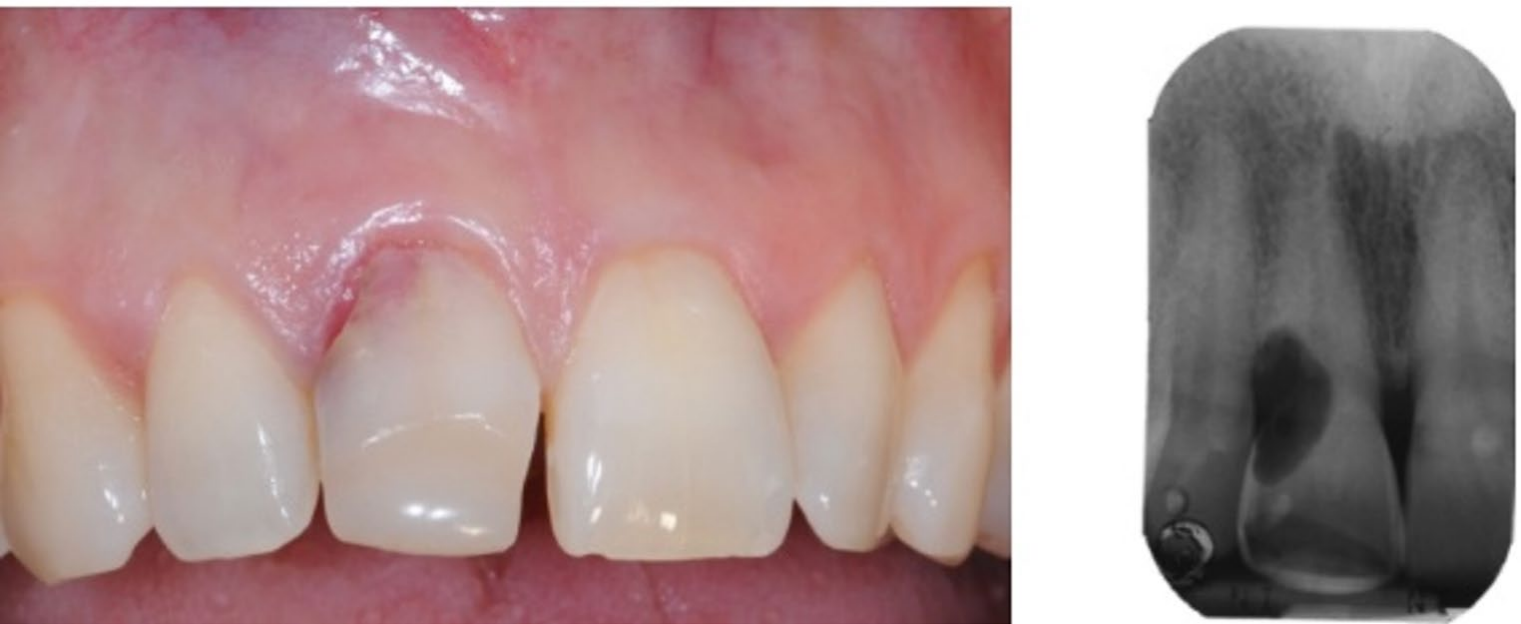
Initial condition and initial X-Ray

**Fig. 7:**
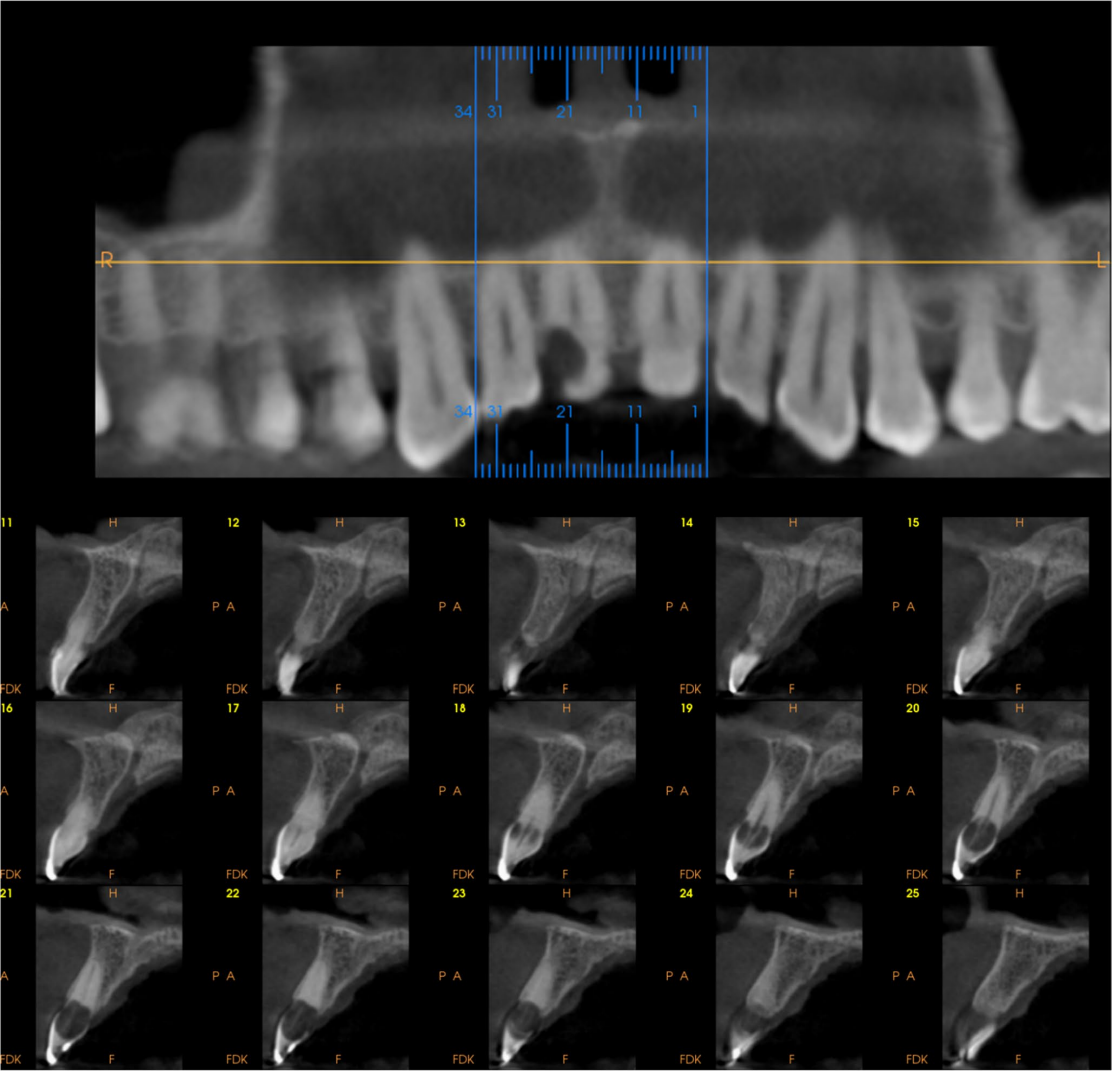
Preoperatory CBCT

A 58-year-old female patient presented to the dental emergency service at AOUIVR (Azienda Ospedaliera Universitaria Integrata di Verona, Verona, Italy) with persistent, increasing pulsating pain in the upper anterior region. She reported no relevant medical history, no current medication use, and no known allergies. Additionally, she met all inclusion criteria. After obtaining informed consent for the clinical examination, study enrolment, and the use of photographs for publication purposes, an intraoral inspection and radiographic evaluation (X-ray) were performed. Clinical and radiological findings confirmed a diagnosis of extensive external root resorption (Fig. 6). Based on these findings, CBCT data were acquired, and an immediate Implant Placement (IIP) procedure was planned (Fig. 7).

**Fig. 8:**
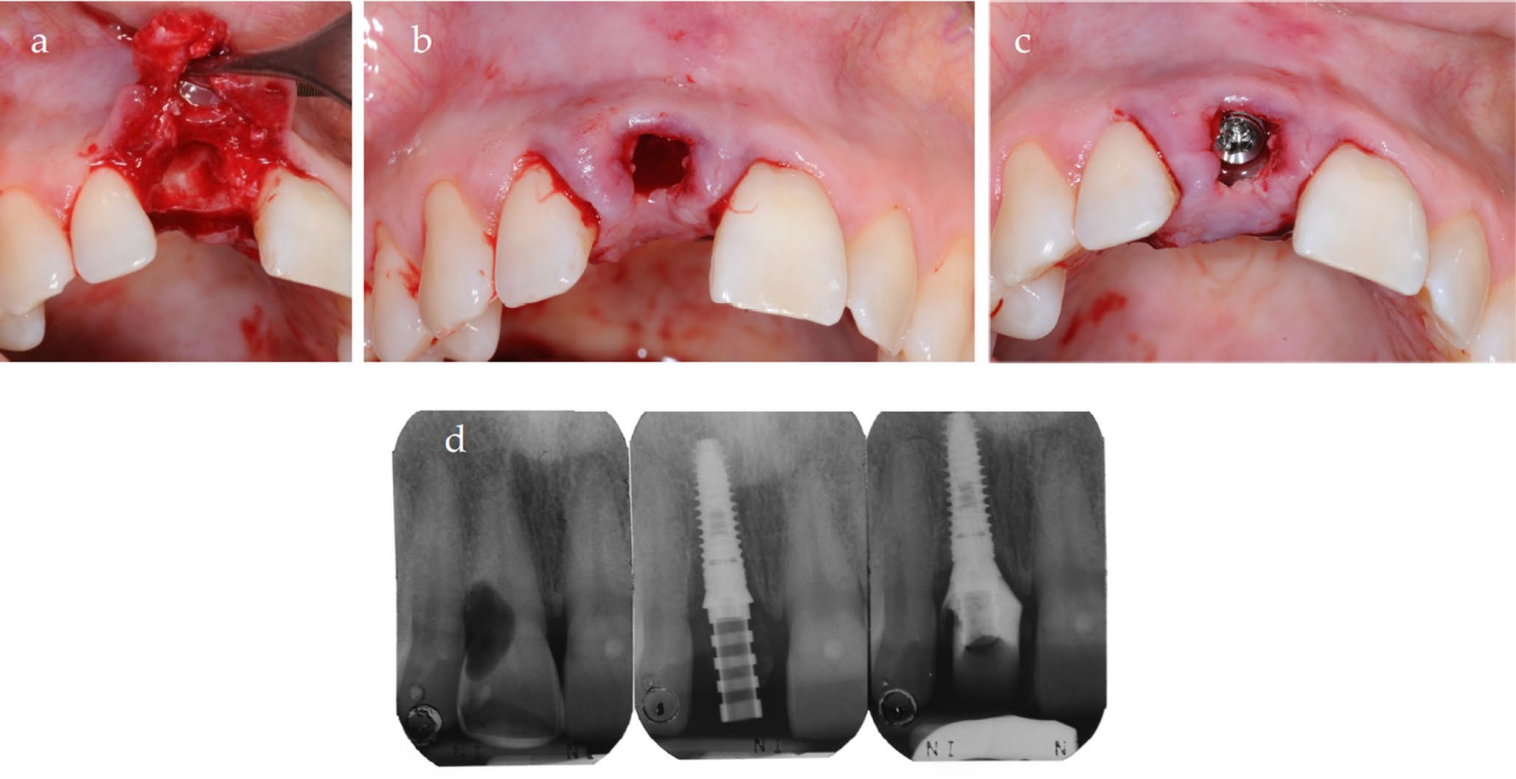
**a** the LID flap is elevated, and the tooth is removed. **b** the interlock feature of the flap is shown. **c** the implant is positioned in situ and the flap is sutured

**Fig. 9:**
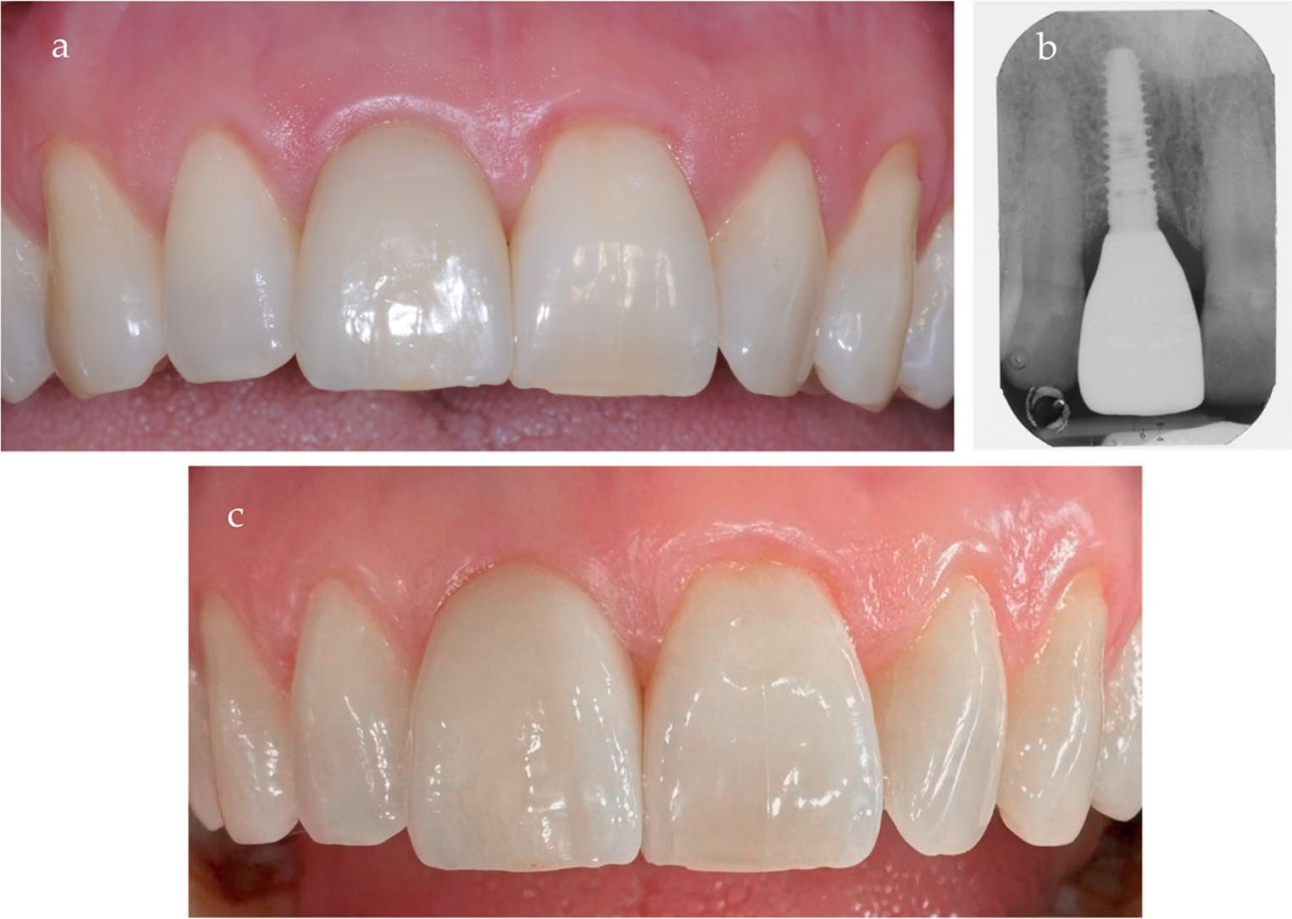
**a** Final restoration. **b** Final restoration X-Ray. **c** 1 year follow-up

The surgery was performed following the procedures outlined in the previous sections. Clinical evaluation confirmed that the buccal plate was essentially intact at the time of surgery, as partially visible in Fig. 8a and confirmed by socket sounding following the extraction. An implant (DentalTech, Misinto, Italy) measuring 13 mm in length and 4.25 mm in diameter was placed (Fig. 8). An immediate provisional restoration was delivered on the same day. Postoperative follow-ups were conducted according to the study protocol at 3, 7, 14, and 30 days after surgery, with sutures removed two weeks postoperatively. After a six-month healing period, the final restoration was delivered (Fig. 9a and b). The Pink Esthetic Score (PES) and the Visual Analogue Scale (VAS) were evaluated one year later (Fig. 9c). This case pertains to patient #17, who achieved a PES score of 13 and a VAS of 10.

## Discussion

Immediate Implant Placement (IIP) within the anterior maxilla represents a complex procedure, necessitating comprehensive preoperative evaluation to achieve proper esthetic outcomes [3, 31]. In addressing preoperative concerns, Kan et al. [32] have proposed a classification of the sagittal root position within the alveolar socket; Shi et al. [33] have highlighted the relationship between the root angle of maxillary incisors and various aspects of surrounding bone anatomy, including coronobuccal, coronopalatal, apicobuccal, palatal, and below apex bone thicknesses. Complementarily, Wychowański et al. [34] have introduced a novel parametric evaluation for preoperatively estimating the feasibility of IIP in the anterior maxilla, particularly emphasizing a flapless approach.

Current literature [31, 35, 36] underscores the importance of three axial dimensions in implant placement within the esthetic zone: mesiodistal, buccopalatal, and coronoapical. Each dimension is characterized by specific ‘safe’ and ‘risk’ zones, determined by the likelihood of suboptimal outcomes. The selection of flapless procedures for Immediate Implant Placement (IIP) is advised limited to specific optimal clinical scenarios [20, 37, 38], even if it is less invasive [39] and can lead to better esthetic results in the short-term [37, 40].

Such a recommendation derives from the complexity and variability of factors influencing successful implant esthetics. Flapless techniques are not suitable for all cases, particularly those requiring hard tissue grafting and soft tissue enhancements for suboptimal starting conditions (thin gingival biotype or buccal bone width ≤ 1 mm). On this topic, flapless procedures may allow for socket wedging when the jumping distance is greater than 2 mm [38, 41–45], attenuating the physiological bone dimensional changes that typically follow tooth extraction, but they interfere with major grafting and soft-tissue enhancing procedures, precluding further stabilization [46].

Considering the multifaceted aspects of Immediate Implant Placement (IIP), especially in the esthetic zone, the clinician is often inclined to opt for a flap elevation procedure when inserting implants into fresh extraction sockets. The primary goal in such scenarios remains the maximization of esthetic outcomes and thus providing a flap that maintains satisfactory esthetics while improving the feasibility of IIPs would be valuable. A paper by Hutchens et al. [12] has analysed different flap designs for IIP and highlighted advantages and limitations of each. Concerning flapless surgery, it is stated [47] that it removes the need for incisions and results in less postoperative pain, but it also significantly lowers visibility and grafting possibilities. Common designs include the papilla-sparing incisions, triangular flap, trapezoidal flap, and vestibular incision. Among these design options for implant placement in the esthetic area the papillae-sparing incisions are one of the most appreciated in terms of low invasiveness and esthetic outcomes [48], but few literature focuses on their adoption in Immediate Implant Placement (IIP).

Various papillae-sparing approaches have been proposed for second stage surgery in the esthetic zone, with the most widespread being the U-shaped technique and its variations [13]. This technique involves a slightly palatal horizontal crestal incision, stopping 1 mm before adjacent teeth, along with two vertical incisions extending buccally and palatally to preserve papillae. The U-shaped flap design align with findings by Gomez-Roman et al. [49], who noted that avoiding papilla incisions can significantly reduce bone resorption during healing, with a maximum reduction of 1 mm compared to 3.5 mm with other methods. This preservation is crucial for maintaining the interproximal bone level, allowing to accommodate a distance to the contact point of less than 5 mm, thereby ensuring support for a fully developed papilla [50].

The LID flap design aims to retain the benefits of traditional papillae-involving flap elevation, such as improved surgical visibility and access, while concurrently emulating the esthetic benefits typically associated with flapless procedures and papillae-sparing incisions. The foundation for such a flap design is endorsed by newly introduced concept on papillae vascularization. Furthermore, it differs from the previously cited adaptation of the multiple coronally advanced envelope flap [9], for both extending palatal visibility to further refine implant positioning during non-guided implant placement and avoiding any sectioning of the papillae.

Concerning papillae vascularity, a recent cadaver study by Shahbazi et al. [16] investigated the vascularization of the maxillary vestibule and gingiva: it was found that anastomoses between the vertical branches of the mucosa deriving from the superior labial artery (SLA) and vertical branches deriving from the infraorbital artery (IOA) contribute to papillae vascular supply. Another study by Mikecs [15] occluded a 1.5 mm area at the mucogingival line and found “significant” ischemia at the papilla, reporting that the main blood supply originates from gingival vessels branched by the SLA and its only after a damage (flap/incision) that alveolar and periodontal vessels may become relevant by collateralization. The LID technique preserves such gingival vessels by avoiding any kind of splitting incision on papillae and discouraging any incision on the buccal side. Based on these findings, the LID flap aims to preserve the periosteal layer, elevating papillae at split-thickness; while this is predictable in thick gingival biotype, the risk of damaging the flap increases in thin ones and thus the authors recommend it only in selected cases and/or under microscopic guidance.

In terms of esthetics, the LID flap design avoids vertical incisions on the buccal aspect, and the buccal soft tissue is barely elevated during the procedure. This approach reduces the risk of soft tissue recession during healing, a critical factor in achieving high esthetic outcomes [51]. Furthermore, this technique offers great adaptability, allowing it to be tailored to a wide range of clinical scenarios without the need for multiple, distinct flap designs; in cases of soft tissue enhancement, as often required by IIP [50], an envelope flap can be created at the buccal aspect of the implant site without interfering incisions. Moreover, the LID design reduces the risk of buccal wall fractures during the extraction of the failing tooth, granting optimal visibility on the root surface and thus making immediate implant placement more predictable [52].

It should be noted that the LID design fails to address two key concerns related to IIP. In borderline cases requiring extensive regeneration alongside implant placement, the flap design does not allow sufficient flap mobilization, often necessitating a buccal vertical release incision. Additionally, a drawback of the proposed flap design is its impact on papilla management in thin gingival biotypes. The clinician must weigh the potential complications against the benefits of the proposed flap design and, in certain situations, reverting to traditional flap techniques may be a more prudent approach.

In relation to study design limitations, the authors acknowledge that while the LID flap was designed to preserve critical vascular pathways described in recent anatomical studies [15, 16], no objective assessment of gingival blood supply was performed in this case series, nor was a direct comparison with a control group included. As these elements represent essential foundations for establishing causality, any assumptions regarding improved vascularity must be interpreted as theoretical. Validation through future comparative studies or perfusion-based assessments will be necessary to substantiate this hypothesis.

On the other hand, the Pink Esthetic Score (PES) archived in this paper, with a mean value of 12.35 ± 0.99 on 20 implants, is to be considered, in authors‘ opinion, a good mean result, as Cosyn reported optimal being ≥ 12 and unsatisfactory ≤ 8 [53] and other authors [54] consider the PES being “good” when ≥ 10. It is comparable to the ones reported by literature [50, 53–55], but with significantly less variability among patients. Furthermore, these findings indicate that while the LID flap design shows promising esthetic outcomes, it may still be limited in cases with poor initial conditions. The high VAS scores (9.15 ± 0.75) align with previous findings in the literature [56], where Immediate Implant Placement (IIP) combined with provisionalization and minimal soft tissue trauma led to improved psychosocial acceptance and comfort during the healing phase.

## Conclusion

In the present study, the authors present the LID flap design for Immediate Implant Placement (IIP). The newly proposed flap aims to effectively combine the esthetic advantages of flapless procedures with the predictability of vestibular flap elevation. This approach shows promising result, particularly in enabling atraumatic tooth extractions and implant placement without vestibular elevation. The preliminary results of the present retrospective case series are encouraging, indicated by a high Pink Esthetic Score (PES) of 12.35 ± 0.99 one year after final prosthetic delivery and minimal postoperative complications. However, the authors emphasize the need for a structured clinical trial to validate these results and assess the design’s effectiveness across diverse clinical scenarios.

## Supplementary Information

Below is the link to the electronic supplementary material.

ESM 1 (PDF 145 KB)

## Data Availability

Data is provided within the manuscript.
