# Supplementary material for: The low invasiveness design (LID) flap in immediate implant placement: a 20-patient case series on a novel flap design based on new insights into papilla vascularization
Source: Clin Oral Investig. 2025 Aug 12;29(9):410. doi: 10.1007/s00784-025-06496-x (PMC12343692; doi:10.1007/s00784-025-06496-x)
Supplement: Supplementary file 1 — (PDF 145 KB) [file 784_2025_6496_MOESM1_ESM.pdf]

COMITATO ETICO TERRITORIALE AREA SUD OVEST VENETO

Istituito con Decreto del Direttore Generale dell'Area Sanità e Sociale n. 330 del 29 marzo 2023
con sede operativa c/o Azienda Ospedaliera Universitaria Integrata Verona
P.le A. Stefani, 1 - 37126 Verona - Tel. 045 8123236 - Fax 045 8123177
e-mail: comitatoetico@aovr.veneto.it
PEC: comitatoetico.aovr@pecveneto.it

Verona, 24/07/2024

To whom it may concern

Subject: Retrospective observational studies

Pursuant to the applicable regulations on personal data protection and clinical trials, for retrospective observational studies, the Ethics Committee merely takes note of the study, rather than evaluating it, in cases where such a study involves the use of anonymous data or where consent to the processing of personal data is collected from all the participants in the research. The Ethics Committe issues an opinion on the research project only in the remaining cases.

Prof. Roberto Leone

President of the Territorial Ethics Committee "*Area Sud Ovest Veneto*"

Roberto Leone
